# Acute Diarrhea in a Tertiary Emergency Department: From Readmission Determinants to Antibiotic Prescription

**DOI:** 10.3390/antibiotics13090891

**Published:** 2024-09-16

**Authors:** Marcello Covino, Antonella Gallo, Fiammetta Maria Rognoni, Maria Caterina Parlangeli, Benedetta Simeoni, Francesco Franceschi, Francesco Landi, Massimo Montalto

**Affiliations:** 1 Department of Emergency Medicine, Fondazione Policlinico Universitario “A. Gemelli” IRCCS, 00168 Rome, Italy; marcello.covino@policlinicogemelli.it (M.C.); benedetta.simeoni@policlinicogemelli.it (B.S.); francesco.franceschi@policlinicogemelli.it (F.F.); 2 Department of Emergency Medicine, Università Cattolica del Sacro Cuore, 00168 Rome, Italy; 3 Department of Geriatrics, Orthopedics and Rheumatology, Fondazione Policlinico Universitario “A. Gemelli”, IRCCS, 00168 Rome, Italy; francesco.landi@unicatt.it (F.L.); massimo.montalto@unicatt.it (M.M.); 4 School of Internal Medicine, Università Cattolica del Sacro Cuore, 00168 Rome, Italy; fiammettamaria.rognoni01@icatt.it (F.M.R.); maria.parlangeli01@icatt.it (M.C.P.); 5 Department of Geriatrics, Orthopedics and Rheumatology, Università Cattolica del Sacro Cuore, 00168 Rome, Italy

**Keywords:** acute diarrhea, emergency department, antibiotics, probiotics, readmission

## Abstract

Acute diarrhea represents a major public health issue, and the management of adult patients admitted to the emergency department (ED) for this problem is still challenging. In a retrospective analysis on more than 20,000 patients visiting a tertiary ED for acute diarrhea and then being discharged home, we found that age > 65 years, onset of symptoms > 24 h since ED admission, refusal of hospitalization, and a history of chronic renal and liver diseases were independently associated with ED readmission for abdominal symptoms within 7 days. In the younger group, the presence of comorbidities significantly impacted on ED readmission, while fever and alteration of serum creatinine were the main determinants in the older group. Antibiotics were prescribed in about 25% of patients, although diarrhea etiology (viral or bacterial) was usually not available. According to international guidelines, fluoroquinolones were the most prescribed class, showing an inverse correlation to ED readmission. However, β-lactams and probiotics were also commonly prescribed; the latter were independently correlated to ED readmission in the elderly group. A comprehensive, guideline-based approach, including a detailed clinical history and laboratory and comorbidity assessment, should be encouraged to support physicians in the management of different age subgroups of adults admitted to the ED for acute diarrhea.

## 1. Introduction

Acute diarrhea is a major public health issue and an important cause of outpatient visits and hospitalizations [1]. It represents one of the top ten causes of death worldwide, contributing to over 1.7 million deaths among all ages and all over the world [2]. In resource-limited settings, it is the third cause of death among children younger than five years old [2,3]. Conversely, in resource-abundant settings, although associated with minor mortality, diarrheal disease frequently causes a loss of quality of life, contributing to the loss of 45.5 million disability-adjusted life years (DALYs) [3]. Furthermore, the related costs are significant; according to the Centers for Disease Control and Prevention, 47.8 million cases occur annually in the United States, at an approximated cost of 150 million US dollars to the healthcare economy [1].

Acute diarrhea is defined by the World Health Organization (WHO) as the presence of three or more loose or liquid stools in a 24 h period for 14 days or fewer [4]; according to other definitions, there is no minimum number of discharges, as acute diarrhea can be defined when there is an increased number of stools of reduced consistency compared to the normal condition lasting less than 14 days [1]. The main cause of acute diarrhea is infection [1]; in most cases, it is of viral origin, and among these, despite the vaccination program adopted by a growing number of countries [5], *Rotavirus* remains the leading etiology among all age groups worldwide [2]. In developed countries, *Norovirus* is the most frequent cause [6,7], although bacterial etiologies, in particular *Campylobacter*, enteropathogenic *Escherichia coli*, and enteroaggregative *E. coli (EAEC)*, appear to have a more severe clinical presentation; lastly, *Clostridioides difficile* is emerging as a cause of community-acquired diarrheal cases, mainly in high-income countries [6]. Moreover, the quality of consumed water and foodborne transmission may play a significant role in acute diarrhea prevalence according to patients’ geographical provenance.

To the best of our knowledge, the American College of Gastroenterology (ACG) clinical guidelines [1] and the Infectious Diseases Society of America (IDSA) clinical guidelines [8] represent the most recent guidelines on the management of adults with acute diarrhea. According to them, regardless of the etiology, management should start with general measures such as fluid repletion and nutrition maintenance. The habitual use of antibiotics should be deterred from, as in most cases, acute diarrhea has a viral etiology, and therefore does not benefit from the use of antibiotics either in terms of duration or in terms of symptoms. However, empiric and targeted antibiotic therapy is appropriate in patients with severe disease, with symptoms and signs suggestive of invasive bacterial infection or at high risk of complications (age ≥ 65 years, cardiac disease, immunocompromised condition, inflammatory bowel disease, pregnancy) [1,8]. When the decision to treat acute diarrhea has been made, azithromycin or a fluoroquinolone are the antibiotics of choice depending on the local susceptibility patterns and the patient’s travel history [1,8]. 

However, the management of adults with acute diarrhea still remains a challenge for doctors, who must weigh the risks and benefits of each approach. This is especially the case for physicians working in the emergency department (ED), who are often asked to make decisions in a very limited period of time. A validated and shared approach to manage patients with acute diarrhea in this setting is still lacking and there are few real-life studies focused on this topic [9,10,11]. In particular, they all shed light on disorderly management, often non-compliant with current guidelines, which may result in injury to the patient (possible under- or over-treatment; development of antibiotic resistance) and to the healthcare system (increased costs; increased number of hospitalizations; early readmission) [9,10,11]. 

Our large retrospective study performed in a tertiary ED at a university center aimed to evaluate the determinants, including antibiotic therapy, related to early (within seven days) readmission for abdominal symptoms in a cohort of patients with acute diarrhea.

## 2. Materials and Methods

### 2.1. Patients and Setting 

This is a retrospective study conducted in the tertiary ED of the Fondazione Policlinico Universitario A. Gemelli IRCCS of Rome, Italy. In the analysis, we included all adults (aged > 18 years) who accessed the ED for acute diarrhea between 2015 and 2022 and were then discharged home without hospitalization. The majority of people attending our ED come from Central Italy, less commonly from the South and even less so from North Italy. Our hospital is the largest teaching hospital in Rome and a referral center for COVID-19 in Central Italy. Our ED has a catchment area of 1.8 M inhabitants, with an average of 80 k access/year.

We excluded patients with neoplastic disease in active treatment, since it is conceivable that in those patients diarrhea may usually be caused by other mechanisms than the infectious one, mainly drug toxicity [12,13], and patients with a confirmed SARS-CoV-2 infection (as documented by PCR or antigenic test on nasopharyngeal swab performed at admission to the ED). This study was conducted according to the principles expressed in the Declaration of Helsinki and its later amendments and was approved by the local Institutional Review Board (#0025817/22 on 3 August 2022).

### 2.2. Data Collection

Data were retrospectively collected from the computerized clinical records of our institution and included in an electronic database.

We collected the following variables for each subject:

- Characteristics of ED access, including self-reported or transferred by the emergency medical system (EMS); triage code assigned, length of stay in the ED (hours);
- Patient demographics, including age and sex;
- Onset of diarrhea (before or after 24 h since ED admission);
- Refusal of hospitalization;
- Associated complains at ED admission, including fever over >37.5 °C, vomiting, nausea, abdominal pain, chest pain, syncope, melena, rectorrhagia, belching/hiccups, malaise;
- Clinical history, including the presence of diverticulosis, gastroesophageal reflux disease (GERD), inflammatory bowel disease (IBD), cardiac disease, chronic obstructive pulmonary disease (COPD), diabetes, chronic renal failure, liver disease;
- Laboratory evaluation (if obtained), including white blood count (WBC), C-reactive protein (CRP) value, hemoglobin (Hb) values, serum creatinine, and serum procalcitonin (if obtained). Laboratory tests were available 24 h/day in our institution in the study period;
- Prescription of any antibiotic at discharge. Antibiotic prescriptions were grouped for into non-absorbable and absorbable antibiotics further analysis. The search list of absorbable antibiotics includes amoxicillin, amoxicillin/clavulanic acid (Am/Cl), ceftriaxone, azithromycin, clarithromycin, ciprofloxacin, levofloxacin, fluoroquinolones, metronidazole, vancomycin, and doxycycline;
- Prescription of probiotics at discharge, alone or in combination with antibiotics.

### 2.3. Outcome Measures

As the primary outcome, we evaluated the determinants of readmission to the ED for abdominal symptoms within seven days in patients discharged from the ED at index admission.

As a secondary endpoint, we analyzed the rate and type of antibiotic/probiotic prescription at discharge.

Both endpoints were evaluated in the whole adult population and in patients under and over 65 years. 

### 2.4. Statistical Analyses

Continuous variables were reported as medians [interquartile ranges] and were compared at univariate analysis by the Mann–Whitney *U* test or Kruskal–Wallis test in case of three or more groups. Categorical variables were reported as absolute numbers (percentages) and were compared by the chi-squared test (with Fisher’s test if appropriate).

Only patients discharged from the ED at the index visit were included in the analysis. The factors showing a significant association with the readmission to the ED within seven days were entered into a logistic regression model in order to identify independent predictors of readmission. 

Both univariate and multivariate analyses were carried out in the whole population and separately in the patients divided into two groups according to age: those aged 18–64 years and those ≥65 years old. 

Multiple multivariate models were obtained for “any antibiotic, antibiotic+probiotic and probiotic”. 

Multivariate association of factors with the study endpoints was expressed as odds ratio (OR) [95% confidence interval]. A two-sided *p* ≤ 0.05 was considered significant. Data were analyzed by SPSS (Version 25).

## 3. Results

### 3.1. Study Population

In the study period, 20,581 adults were admitted to our ED for acute diarrhea and then discharged home and were included in the analyses (Table 1).

The median age was 48 years (IQR 33–66), and 8107 patients (39.4%) were males. In particular, 15,063 (73.2%) were younger than 65 years, while 5518 (22.8%) were older than 65 years. Demographic data and the main clinical data are reported in Table 1, both relative to the total cohort and to the different subgroups stratified age. Older people were more frequently assigned both an emergency and urgency triage code at ED admission compared to younger subjects, who were more frequently assigned a non-emergent triage code (*p* < 0.001). Access to EMS was significantly more common for older people compared to younger subjects (26.2% vs. 12.8%, *p* < 0.001). 

The main associated complaints were fever, vomiting, nausea, and abdominal pain. In particular, fever, nausea, and abdominal pain were significantly more frequent in the younger group (*p* < 0.001), unlike vomiting, which was significantly more frequent in the older group (*p* < 0.05). 

Non-abdominal complains (i.e., chest pain) and concomitant gastrointestinal bleeding were more frequent in the older group (*p* < 0.001). 

All the laboratory parameters were significantly worse in the older group (*p* < 0.05 for all comparisons). Also, the rate of comorbidities was significantly higher in the older group (*p* < 0.001) for all comparisons.

### 3.2. Determinants of ED Readmission within Seven Days in the Total Cohort

Table 2 shows the factors that were or were not associated with ED readmission for abdominal symptoms within seven days.

Overall, in univariate analysis, older age, lack of access by EMS, onset of symptoms > 24 h since ED admission, and refusal of hospitalization were the demographic/anamnestic factors associated with ED readmission (*p* < 0.05 for each of comparisons). Patients showing lower levels of Hb at ED admission were more likely to be readmitted for abdominal symptoms within seven days (*p* < 0.001). Other clinical and laboratory parameters that were or were not associated with ED readmission in univariate analysis are detailed in Table 2. History of chronic renal failure and liver diseases were significantly associated with ED readmission in the total cohort (*p* < 0.05). Finally, univariate analysis showed that fluoroquinolone (in particular, ciprofloxacin) prescription at discharge was inversely associated with ED readmission, unlike ceftriaxone prescription at discharge (*p* < 0.05). 

When entered into a multivariate logistic regression model, age > 65 years (OR 1.3, *p* < 0.001), onset of symptoms > 24 h since ED admission (OR 1.2, *p* < 0.001), refusal of hospitalization (OR 1.7, *p* < 0.001), and history of chronic renal failure (OR 1.6, *p* < 0.05) and liver diseases (OR 1.7, *p* < 0.05) were independently associated with ED readmission. 

### 3.3. Determinants of ED Readmission within Seven Days According to Age Subgroups

Table 3 and Table 4 show factors associated with ED readmission for abdominal symptoms within seven days in the younger and older subgroups, respectively. 

In particular, in the younger subgroup, univariate analysis showed that lack of access by EMS (*p* < 0.05), onset of symptoms > 24 h since ED admission (*p* < 0.01), and refusal of hospitalization were the demographic/anamnestic factors associated with ED readmission. Other clinical and laboratory parameters analyzed by univariate analysis are detailed on Table 3. 

History of chronic renal failure (*p* < 0.01), liver diseases (*p* < 0.01), and dementia (*p* < 0.01) showed a significant correlation with ED readmission within seven days. Also, antibiotic prescription alone (in particular, fluoroquinolones and ciprofloxacin) at discharge was inversely associated with ED readmission within 7 days (*p* < 0.05), unlike amoxicillin/ clavulanic acid and ceftriaxone (*p* < 0.05). 

When entered into a multivariate logistic regression model, onset of symptoms > 24 h since ED admission (OR 1.2, *p* < 0.05), refusal of hospitalization (OR 1.8, *p* < 0.001), and history of chronic renal failure (OR 2.4, *p* < 0.01), liver diseases (OR 2.2, *p* < 0.01), and dementia (OR 6.0, *p* < 0.01) were independently associated with ED readmission.

The rate of ED readmission was significantly higher in the spring/summer period vs. autumn/winter (6.4% vs. 5.5%; *p* < 0.01). 

Conversely, in the older subgroup, univariate analysis showed that the male sex (*p* < 0.05), triage code (*p* < 0.05), lack of access to EMS (*p* < 0.05), onset of symptoms > 24 h since ED admission (*p* < 0.01), and refusal of hospitalization (*p* < 0.01) were the demographic/anamnestic factors associated with ED readmission, as was a concomitant occurrence of fever at ED admission (*p* < 0.05). Other clinical and laboratory parameters that were or were not associated with ED readmission on univariate analyses are detailed in Table 4. 

History of diverticulosis (*p* < 0.01) also showed a significant inverse correlation with ED readmission in univariate analysis. 

When entered into a multivariate logistic regression model, the clinical findings and symptoms independently associated with ED readmission were the male sex (OR 1.3, *p* < 0.05), triage code (OR 1.7 and 2.03, *p* = ns), onset of symptoms > 24 h since ED admission (OR 1.3, *p* < 0.05), refusal of hospitalization (OR 1.4, *p* < 0.05), fever (OR 1.3, *p* < 0.05), and probiotic prescription alone (OR 1.1, *p* = 0.8)

### 3.4. Antibiotic Prescription

**Figure 1:**
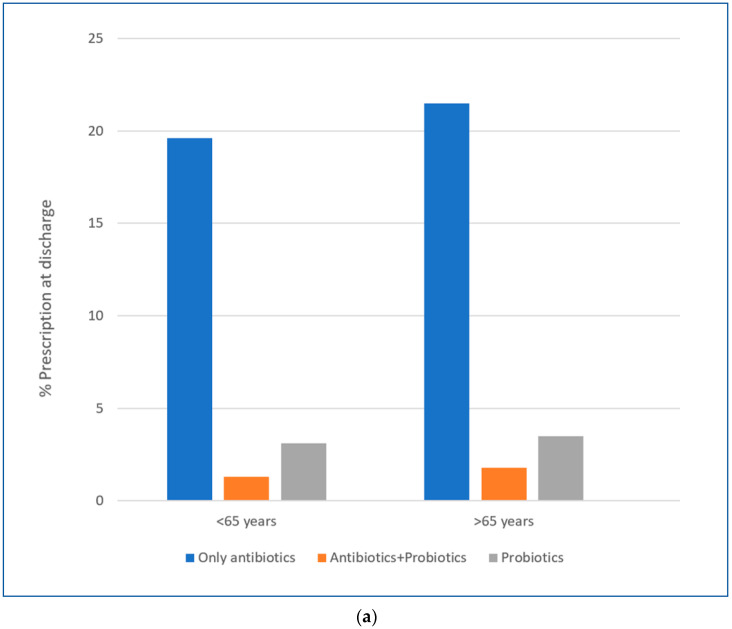

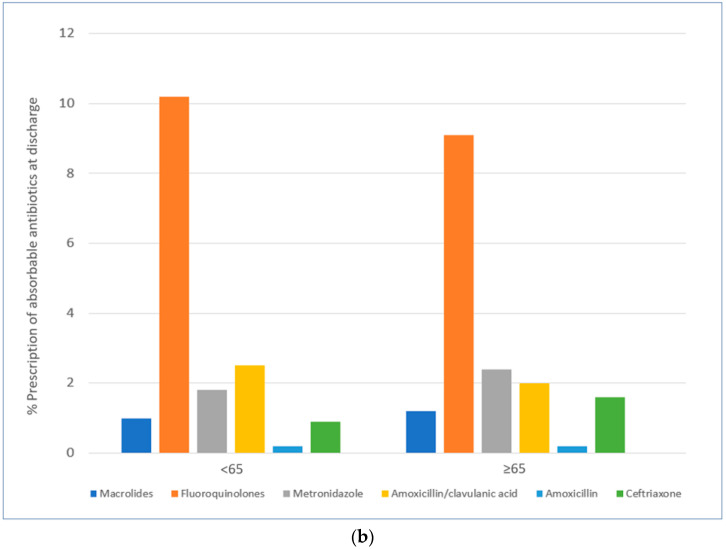
(**a**) Rate of antibiotic/probiotic prescriptions in the study population divided by age subgroups. (**b**) Rate of the most common absorbable antibiotic prescriptions in in the study population divided by age subgroups.

Overall, more than twenty per cent of the evaluated patients received an antibiotic prescription, alone or in combination with probiotics, significantly more frequently in the younger group compared to the older group (23.7% vs. 21.9%). Non-absorbable antibiotics were prescribed in 2012 patients (9.1%) and probiotics in 1078 (5.2%) patients. Non-absorbable antibiotics were more commonly prescribed in the older group (9.6 vs. 9.3%), although this difference was not significant (*p* = 0.08). Probiotics were more commonly prescribed in the younger group, both in association with antibiotics (1.8% vs. 1.6%, *p* = 0.065) and alone (3.8% vs. 2.5%, *p* < 0.001) (Table 1, Figure 1a).

Fluoroquinolones were the most prescribed class of absorbable antibiotic (2044 patients, 9.9% of all prescriptions) in both groups (Figure 1b), although they were more frequently prescribed to younger subjects compared to older ones (*p* < 0.05). Conversely, metronidazole prescription at discharge was more common in older compared to younger patients (*p* < 0.05). When available, the duration of suggested antibiotic therapy ranged from one to five days, according to the clinician’s judgment and current guidelines.

Stratification by triage codes did not correlate with absorbable antibiotic prescription, both in the whole cohort (*p* = 0.18) and in the two age subgroups (*p* = 0.97 in the younger groups and *p* = 0.60 in the older group). Conversely, prescription of non-absorbable antibiotics was significantly correlated with non-urgent triage codes at ED admission (*p* < 0.001 for all groups). The same correlation was reported between probiotic prescription and non-urgent triage code at ED admission (*p* < 0.001 for all groups).

No seasonal (spring/summer period vs. autumn/winter) differences were reported in the percentages of absorbable antibiotic, non-absorbable antibiotic, and probiotic prescriptions (*p* = ns for each comparison).

Further details on the study population are reported in Table 1.

## 4. Discussion

Acute diarrhea represents a common reason for admission to the ED. In our retrospective analysis, we found that, even not including those subjects who were further hospitalized, more than 20,000 people visited our tertiary-center ED for this complaint within eight years.

As is widely known, the ED represents a very particular context. Legal risks [14], lack of access to comprehensive diagnostics and clinical background, high flows of patients [15], lack of dedicated follow-up paths, and patient expectations [10,16] make physicians’ evaluation and decision processes more difficult. Risk stratification, choice of discharge vs. hospitalization, and appropriate discharge indications may represent a challenge for many physicians facing a lot of different conditions, including acute diarrhea.

Epidemiological studies show us that, although the incidence of acute diarrhea in the subgroup of adults (>65 years) is about half of the incidence in the subgroup of children (<5 years old), the mortality rate in adults is almost three times greater than in children [2]. This suggests that global population aging has increased the diarrhea burden in this age group. Moreover, the main increase in diarrhea mortality among people older than 70 years has occurred in high-income countries, including the USA [2]. This condition can surely in part be explained by the burden of *C. difficile*, which, in its hyper-virulent strains, affects not only hospitalized people with recent antibiotic exposure, as traditionally considered, but also individuals without previous exposure to antibiotics [17,18,19]. However, the impact of comorbidities and other risk factors has not been completely elucidated.

In our study, we found that older age represents an independent determinant for readmission to the ED for abdominal symptom complaints. It is likely that the higher rate of comorbidities we reported, typical of this particular subgroup, significantly impacted disease outcomes. However, we have to consider that older people were more frequently admitted to the wards rather than discharged, thus limiting the analysis for this group of patients.

Interestingly, we found that the burden of comorbidities also plays a relevant role in the younger group, representing one of the most significant determinants in ED readmission. The presence of pre-existing comorbidities in the younger group, such as chronic renal and liver disease and, most of all, dementia, showed an independent significant impact on disease outcome. This result clearly supports the concept that, besides age, a comprehensive assessment of frailty and functional status at ED admission may undoubtedly help physicians in risk stratification and decision-making in such a difficult clinical setting.

Because of the retrospective design of this study, we are not able to provide data about the frailty assessment of the whole population; instead, we know their comorbidity status. Although this represents an incomplete assessment, the interplay between frailty and comorbidities is undoubtedly close, as frail patients often have comorbidities, and comorbid diseases may lead, at least additively, to the development of frailty.

As the population ages, these concepts become increasingly relevant in providing health care to the population. Nowadays, it has been clearly established that frail individuals experience higher rates of adverse outcomes than others of the same chronological age [20,21,22,23]. Therefore, early assessment of frailty and functional status, regardless of age, may become essential in order to identify those patients who are most vulnerable, helping clinicians in discriminating which interventions will be more likely to be beneficial and which may be more harmful to particular individuals.

Additionally, in the subgroup of older people, globally characterized by a high comorbidity rate, we found that fever and serum creatinine represent independent parameters linked to ED readmission, perhaps indicative of more vulnerable patients unable to restore fluid depletion. Ion imbalance and dehydration were largely described as risk factors for a worse disease outcome until death. We found that the rate of ED readmission was higher in the spring/summer period, perhaps due to dehydration status. In this context, hematocrit values may also represent a parameter of hydration status, mainly in older subjects. Although we did not include them in our analysis, unlike serum creatinine, they would certainly be of great importance, particularly in the risk stratification and management of complex patients.

Overall, we found that a large proportion of adults, as high as one in four, were discharged from the ED with antibiotics. Among the antibiotic classes, fluoroquinolones were the most prescribed. These data are in line with previous studies [9,10] and with the current guidelines on the management of acute diarrhea in the general adult population [1,8]. In our sample, fluoroquinolone prescription at discharge shows a significant inverse correlation with ED readmission in all the analyzed subgroups, in contrast to other classes of antibiotics, such as β-lactams (amoxicillin/clavulanic acid and ceftriaxone), which are not included in the current guidelines. We were not able to recognize whether prescribing these classes of antibiotics was related to patients’ potential drug allergies or concomitant diseases or to physician preference differing from guideline suggestions. However, our results confirmed that a guideline-driven approach may have a significantly positive impact on disease outcome. We also reported that non-absorbable antibiotics and probiotics were also a common therapeutic option, the latter particularly in younger subjects. It is conceivable to suppose that, in the absence of an etiological diagnosis, these two classes of drugs were considered less harmful and with potential beneficial effects on mild forms of the disease, even if there is no evidence of this in the current guidelines. This finding is also supported by the significant correlation between non-absorbable or probiotic prescription and a less serious clinical presentation at ED admission, as assessed by the triage system. It is interesting to note that, in our study, the prescription of probiotics alone negatively impacts the seven-day readmission to the emergency department in the older subgroup, in contrast to younger subjects. Although we did not stratify our cohort according to disease severity, it is likely that a large number of younger (and not comorbid) people may benefit from less aggressive approaches, or maybe even just general fluid repletion and nutrition maintenance. On the contrary, in older and comorbid people, this strategy may not be sufficient.

Recognizing the etiology of acute diarrhea certainly represents one of the most important challenges for ED physicians. However, while waiting for rapid diagnostic testing methods for enteric pathogens in real practice—which may not always lead to a diagnosis or change in treatment choice—the clinician’s judgment often remains the most relevant instrument, particularly in discriminating between infectious vs. non-infectious diarrhea but also between bacterial and viral infections and, finally, in the decision on whether to start empirical antibiotic therapy or not. This diagnostic gap significantly impacts the consequent best approaches to adopt, especially in a context characterized by a growing antibiotic resistance and global calls to activate proper antimicrobial stewardship programs, including in ED settings [24,25]. Antimicrobial resistance (AMR) represents one of the most significant public health concerns. Despite increased prevention measures for multidrug-resistant organism infection control, Italy remains one of the European countries with the highest levels of AMR [26]. As an example, data from the AR-ISS national antibiotic resistance surveillance system by the Italian National Institute of Health showed that in 2019, the resistance rate of *Escherichia coli* to third-generation cephalosporins was about 30%, whereas the rate of resistance to fluoroquinolones slightly decreased over the last five years (from 44.4% in 2015 to 40.7% in 2019). Regarding Gram-positive bacteria, the rate of methicillin-resistant *Staphylococcus aureus* isolates was about 35%, while the proportion of vancomycin-resistant *Enterococcus faecium* significantly increased in the latest years, to about 22% [26].

We found that older patients received fewer systemic antibiotic prescriptions (and, in particular, fluoroquinolones) at discharge compared to the younger group, possibly due to major concerns regarding antibiotic resistance and, above all, side effects such as alteration of the normal flora with the subsequent risk of antibiotic-associated diarrhea and *C. difficile* infection [17,19]. Even though we cannot discriminate among the rates of different diarrhea etiologies between the two age subgroups, the percentage of associated complains may help in distinguishing the presentation of the same disease in the two different groups. In fact, we reported a higher percentage of fever and abdominal pain in the younger subgroup—perhaps indicative of a percentage of inflammatory bowel disease cases. Conversely, we found that older people were more likely to report the occurrence of gastrointestinal bleeding, perhaps indicative of concomitant associated disease, such as diverticulosis or cancer. Moreover, while fluoroquinolones (and, in particular, ciprofloxacin) were more frequently prescribed to younger subjects, metronidazole was more commonly prescribed to older ones, which may indicate a plausible difference in acute diarrhea etiology between the two age subgroups. Since we analyzed the data of those subjects who were discharged home from the ED, the possibility that any potential empirical antimicrobial therapy was changed after the identification of the etiological agent and its resistance profile is quite low, in consideration of the time necessary to identify the intestinal pathogen. It would be very interesting for large, prospective studies to evaluate the real impact of empirical antimicrobial therapy prescribed in the ED on clinical practice, further than the seven-day readmission period, as well as assessing its appropriateness and the potential consequences related to the adoption, or lack thereof, of guideline-based approaches.

Both a duration of symptoms of more than 24 h and refusal of hospitalization represent two common determinants of ED readmission in the total cohort and in the two age subgroups. Regarding symptom duration, these data are in line with the current guidelines, which include this parameter in the identification of severe disease [1,8]. On the other hand, the interesting data on refusal of hospitalization may highlight the crucial role of physicians’ judgment, which, also with very limited instruments, may guide patients to the best approach.

Therefore, in such a particular context, it is becoming more evident that more comprehensive strategies are needed. New biomarkers and a wider availability of rapid microbiological diagnostic tests could favorably influence clinical practice. However, in addition to this, an improved definition of confirmed/probable viral/bacterial infections, a deeper focus on factors associated with clinical presentations, and physicians’ judgments are each necessary factors to be incorporated into real-world settings and research projects.

Our study has some limitations to address. The retrospective nature of this study is an intrinsic limitation, although our electronic system allowed us to include and analyze a large number of detailed variables. Secondly, we only analyzed records of discharged patients, resulting in possible analysis bias, mainly in the older population, which, due to comorbidities and the possibility of developing complications, is hospitalized more often. Furthermore, we chose to consider ED readmission for abdominal symptoms within seven days as an indirect marker of disease outcome, resulting in missing data of those discharged subjects who did not come back to the same ED, or came back over seven days later, or were managed by general physicians or other doctors. Also, we were not able to trace information about dietary habits, mainly regarding features of consumed water (bottled water or water collected in the street), since most records did not feature this information. Although there were not any significant differences in the regional provenance of our patients, it would undoubtedly be interesting to also analyze these aspects in a prospective study.

In conclusion, although acute diarrhea represents a common reason for admission to the ED, the absence of specific guidelines and the peculiarity of the ED context make the management of these patients a real challenge for physicians. We found that older age represents an independent marker of worse outcomes in the whole population. After stratifying by age subgroups, the burden of comorbidities showed a significant impact in younger subjects, while the alteration of specific clinical and laboratory parameters played a significant role in older patients, perhaps by way of identifying more vulnerable subjects within a group already characterized by a high rate of comorbidities. Although we did not collect the list of chronic drugs for each patient, it is likely that polypharmacy, together with comorbidities, may also play a relevant role in disease outcome. It would be interesting to analyze these data in further evaluations.

Therefore, a comprehensive approach including a detailed clinical history and laboratory and comorbidity assessment can support physicians in the management of different age subgroups of adults admitted to the ED for acute diarrhea.

## Figures and Tables

**Table 1:** Demographic and clinical characteristics and antibiotic prescription of the study population.

Variable	All Patients N 20,581	18–65 Years-Old N 15,063	>65 Years-Old N 5518	*p* Value
Sex (male)	8107 (39.4)	6035 (40.1)	2072 (37.5)	0.001
Age (years)	48 (33–66)	40 (29–51)	76 (70–82)	0.000
Length of stay in the ED (hours)	7.2 [4.6–14.5]	6.7 [4.4–11.8]	9.0 [5.4–23.9]	0.000
Triage code				
Emergency	120 (0.6)	62 (0.4)	58 (1.1)	0.000
Urgent	5548 (27)	3543 (23.5)	2005 (36.3)	
Non-urgent	14,913 (72.5)	11,458 (76.1)	3455 (62.6)	
Access by EMS	3375 (16.4)	1928 (12.8)	1447 (26.2)	0.000
Symptoms > 24 h	10,145 (49.3)	7440 (49.4)	2705 (49)	0.637
Refusal of hospitalization	2305 (11.2)	1644 (10.9)	661 (12)	0.032
Associated complaints				
Fever	7782 (37.8)	6125 (40.7)	1657 (30)	0.000
Vomiting	12,157 (59.1)	8811 (58.5)	3346 (60.6)	0.006
Nausea	7745 (37.6)	5898 (39.2)	1847 (33.5)	0.000
Abdominal pain	12,297 (59.7)	9432 (62.6)	2865 (51.9)	0.000
Chest pain	990 (4.8)	673 (4.5)	317 (5.7)	0.000
Syncope	1791 (8.7)	1099 (7.3)	692 (12.5)	0.000
Melena	190 (0.9)	108 (0.7)	82 (1.5)	0.000
Rectorrhagia	756 (3.7)	510 (3.4)	246 (4.5)	0.000
Belching/hiccups	95 (0.5)	66 (0.4)	29 (0.5)	0.413
Malaise	3134 (15.2)	2114 (14)	1020 (18.5)	0.000
Laboratory parameters				
Hb (g/dL)	13.7 (12.6–14.8)	13.9 (12.9–15)	13.1 (11.8–14.3)	0.000
WBC (cell/mm^3^)	8.58 (6.67–11]	8.6 (6.7–11.1)	8.5 (6.6–10.89)	0.048
Creatinine (mg/dL)	0.79 (0.65–0.98)	0.76 (0.64–0.91)	0.9 (0.71–1.18)	0.000
Blood glucose (mg/dL)	103 (92–120)	100 (89–113)	115 (99–138)	0.000
Procalcitonin (ng/dL)	0.050 (0.05–0.1)	0.05 (0.05–0.09)	0.05 (0.05–0.11)	0.021
CRP (mg/L)	6.9 (1.0–26.9)	5.95 (0.8–24.77)	9.8 (2–34.7)	0.000
Clinical history				
Diverticulosis	904 (4.4)	341 (2.3)	563 (10.2)	0.000
GERD	707 (3.4)	552 (3.7)	1555 (2.8)	0.003
IBD	424 (2.1)	348 (2.3)	76 (1.4)	0.000
Cardiac disease	1392 (6.8)	453 (3)	939 (17)	0.000
COPD	585 (2.8)	221 (1.5)	364 (6.6)	0.000
Diabetes	1461 (7.1)	521 (3.5)	940 (17)	0.000
Chronic renal failure	269 (1.3)	98 (0.7)	171 (3.1)	0.000
Liver disease	219 (1.1)	124 (0.8)	95 (1.7)	0.000
Dementia	252 (1.2)	17 (0.1)	235 (4.3)	0.000
Prescription at discharge				
No antibiotic/probiotic	15,092 (73.3)	10,913 (72.4)	4179 (75.2)	0.045
Only antibiotic	4411 (21.4)	3299 (21.9)	112 (20.3)	0.001
Non-absorbable	2012 (9.1)	1480 (9.3)	532 (9.6)	0.086
Absorbable	2965 (14.4)	2172 (14.4)	793 (14.4)	0.063
Antibiotic (any)+ probiotic	363 (1.8)	276 (1.8)	87 (1.6)	0.065
Only probiotic	715 (3.5)	575 (3.8)	140 (2.5)	0.000
Amoxicillin	44 (0.2)	32 (0.2)	12 (0.2)	0.945
Amoxicillin/clavulanic acid	493 (2.4)	384 (2.5)	109 (2.0)	0.017
Azithromycin	199 (1.0)	139 (0.9)	60 (1.1)	0.285
Clarithromycin	21 (0.1)	15 (0.1)	6 (0.1)	0.855
Ceftriaxone	226 (1.1)	140 (0.9)	86 (1.6)	0.000
Levofloxacin	315 (1.5)	216 (1.4)	99 (1.8)	0.062
Ciprofloxacin	1729 (8.4)	1327 (8.8)	402 (7.3)	0.000
Fluoroquinolones	2044 (9.9)	1543 (10.2)	501 (9.1)	0.013
Metronidazole	404 (2)	269 (1.8)	135 (2.4)	0.002
Vancomycin	21 (0.1)	12 (0.1)	9 (0.2)	0.097
Doxycycline	31 (0.2)	30 (0.2)	1 (0)	0.003

EMS: emergency medical service, Hb: hemoglobin, WBC: white blood cell, CRP: C-reactive protein, GERD: gastroesophageal reflux disease, IBD: inflammatory bowel disease, COPD: chronic obstructive pulmonary disease.

**Table 2:** Determinants of ED readmission for abdominal symptoms within seven days in the total cohort.

Variable	Readmission within 7 Days		Univariate *p* Value	Odds Ratio [95% Confidence Interval]	Multivariate *p* Value
	YES (n. 1216)	NO (n. 19,365)			
Males	506 (41.6)	7601 (39.3)	0.102		
Age (years)	51 (35–69)	48 (33–66)	0.000		
Age (years)			0.000		
18–65	825 (67.8)	14,238 (73.5)			
>65	391 (32.2)	5127 (26.5)		1.329 [1.170–1.510]	0.000
Triage code					
Emergency	5 (0.4)	115 (0.6)			
Urgent	341 (28)	5207 (26.9)	0.504		
Non-urgent	870 (71.5)	14,043 (72.5)			
Access to EMS	166 (13.7)	3209 (16.6)	0.008	0.811 [0.681–0.967]	0.019
Symptoms > 24 h	670 (55.1)	9475 (48.9)	0.000	1.240 [1.099–1.398]	0.000
Length of stay in the ED (hours)	7.1 [4.7–14.3]	7.2 [4.6–14.5]	0.956		
Refusal of hospitalization	206 (16.9)	2099 (10.8)	0.000	1.699 [1.452–1.989]	0.000
Associated complaints					
Fever	461 (37.9)	7321 (37.8)	0.941		
Vomiting	729 (60)	11,428 (59.0)	0.519		
Nausea	455 (37.4)	7290 (37.6)	0.874		
Abdominal pain	748 (61.5)	11,549 (59.6)	0.196		
Chest pain	57 (4.7)	933 (4.8)	0.837		
Syncope	76 (6.3)	1715 (8.9)	0.002	0.732 [0.573–0.935]	0.012
Melena	10 (0.8)	180 (0.9)	0.705		
Rectorrhagia	43 (3.5)	713 (3.7)	0.793		
Belching/hiccups	5 (0.4)	90 (9.5)	0.789		
Malaise	181 (14.9)	2953 (15.2)	0.732		
Laboratory parameters					
Hb (g/dL)	13.5 [12.1–14.5]	13.7 [12.6–14.9]	0.000		
WBC (cell/mm^3^)	8.32 [6.3–10.8]	8.5 [6.7–11.4]	0.054		
Creatinine (mg/dL)	0.81 [0.66–1.03]	0.8 [0.70–1.0]	0.140		
Blood glucose (mg/dL)	102 (90–120.5)	103 (92–120)	0.523		
Procalcitonin (ng/dL)	0.05 [0.05–0.11]	0.05 [0.05–0.10]	0.979		
CRP (mg/L)	8.5 [1.2–28.4]	6.8 [1.2–26.9]	0.449		
Clinical history					
Diverticulosis	46 (3.8)	858 (4.4)	0.285		
GERD	33 (2.7)	674 (3.8)	0.154		
IBD	22 (1.8)	401 (2.1)	0.525		
Cardiac disease	92 (7.6)	1300 (6.7)	0.251		
COPD	42 (3.5)	543 (2.8)	0.186		
Diabetes	100 (8.2)	1361 (7.0)	0.115		
Chronic renal failure	28 (2.3)	241 (1.2)	0.002	1.618 [1.083–2.416]	0.019
Liver disease	23 (1.9)	196 (1.0)	0.004	1.696 [1.093–2.631]	0.018
Dementia	16 (1.3)	236 (1.2)	0.765		
Prescription at discharge					
No antibiotics/probiotics	924 (76.0)	14,168 (73.2)	0.154		
Only antibiotics	238 (19.6)	4173 (21.5)	0.103	0.870 [0.749–1.010]	0.680
Antibiotics+probiotics	16 (1.3)	347 (1.8)	0.221	0.715 [0.430–1.188]	0.195
Only probiotics	38 (3.1)	677(3.5)	0.493	0.897 [0.642–1.254]	0.525
Amoxicillin	4 (0.3)	454 (2.3)	0.370		
Amoxicillin/clavulanic acid	39 (3.2)	188 (1.0)	0.056		
Azithromycin	11 (0.9)	188 (1.0)	0.819		
Clarithromycin	0 (0.0)	21 (0.1)	0.251		
Ceftriaxone	21 (1.7)	205 (1.1)	0.030		
Levofloxacin	18 (1.5)	297 (1.5)	0.883		
Ciprofloxacin	79 (6.5)	1650 (8.5)	0.014		
Fluoroquinolones	97 (8.0)	1947 (10.1)	0.019		
Metronidazole	21 (1.7)	383 (2.0)	0.541		
Vancomycin	2 (0.2)	19 (0.1)	0.482		
Doxycycline	3 (0.2)	28 (0.1)	0.373		

EMS: emergency medical service, Hb: hemoglobin, WBC: white blood cell, CRP: C-reactive protein, GERD: gastroesophageal reflux disease, IBD: inflammatory bowel disease, COPD: chronic obstructive pulmonary disease.

**Table 3:** Determinants of ED readmission within seven days in patients aged 18–65 years old.

Variable	Readmission within 7 Days		Univariate *p* Value	Odds Ratio [95% Confidence Interval]	Multivariate *p* Value
	YES (N 825)	NO (14,238)			
Sex (male)	335 (40.6)	5700 (40)	0.744		
Age (years)	41 (30–52)	40 (29–51)	0.116		
Triage code			0.090		
Emergency	3 (0.4)	59 (0.4)			
Urgent	220 (26.7)	3323 (23.3)			
Non-urgent	602 (73)	10,856 (76.2)			
Access to EMS	83 (10.1)	1845 (13)	0.015	0.781 [0.615–0.991]	0.042
Symptoms > 24 h	445 (53.9)	6995 (49.1)	0.007	1.195 [1.034–1.381]	0.016
Refusal of hospitalization	144 (17.5)	1500 (10.5)	0.000	1.823 [1.509–2.203]	0.000
Length of stay in the ED (hours)	6.7 [4.5–11.8]	6.7 [4.4–11.8]	0.803		
Associated complaints					
Fever	318 (38.5)	5807 (40.8)	0.203		
Vomiting	492 (59.6)	8319 (58.4)	0.494		
Nausea	331 (40.1)	5567 (39.1)	0.559		
Abdominal pain	529 (64.1)	8903 (62.5)	0.358		
Chest pain	39 (4.7)	634 (4.5)	0.711		
Syncope	43 (5.2)	1056 (7.4)	0.018	0.753 [0.547–1.037]	0.083
Melena	5 (0.6)	103 (0.7)	0.698		
Rectorrhagia	33 (4.0)	477 (3.4)	0.316		
Belching/hiccups	2 (0.2)	64 (0.4)	0.381		
Malaise	113 (13.7)	2001 (14.1)	0.774		
Laboratory parameters					
Hb (g/dL)	13.5 [12.3–14.6]	14.1 [12.9–15.1]	0.000		
WBC (cell/mm^3^)	8.53 [6.62–10.77]	8.60 [6.70–11.12]	0.178		
Creatinine (mg/dL)	0.76 [0.63–0.91]	0.76 [0.64–0.91]	0.504		
Blood glucose (mg/dL)	99 (88–114)	100 (89–113)	0.314		
Procalcitonin (ng/dL)	0.05 [0.05–0.10]	0.05 [0.05–0.09]	0.812		
CRP (mg/L)	6.6 [1–26.5]	5.8 [0.8–24.8]	0.600		
Clinical history					
Diverticulosis	19 (2.3)	322 (2.3)	0.938		
GERD	23 (2.8)	529 (3.7)	0.168		
IBD	17 (2.1)	331 (2.3)	0.623		
Cardiac Disease	29 (3.5)	424 (3.6)	0.380		
COPD	15 (1.8)	206 (1.4)	0.388		
Diabetes	35 (4.2)	486 (3.4)	0.205		
Chronic renal failure	13 (1.6)	85 (0.6)	0.001	2.404 [1.322–4.373]	0.004
Liver disease	16 (1.9)	108 (0.8)	0.000	2.236 [1.304–3.835]	0.003
Dementia	4 (0.5)	13 (0.1)	0.001	5.962 [1.931–18.405]	0.002
Prescription at discharge					
No antibiotics/probiotics	630 (76.4)	10,283 (72.2)	0.072		
Only antibiotics	157 (19.0)	3142 (22.1)	0.040	0.825 [0.687–0.991]	0.039
Antibiotics+probiotics	11 (1.3)	265 (1.9)	0.272	0.700 [0.380–1.289]	0.252
Only probiotics	27 (3.3)	548 (3.8)	0.401	0.860 [0.578–1.278]	0.455
Amoxicillin	4 (0.5)	28 (0.2)	0.080		
Amoxicillin/clavulanic acid	31 (3.8)	353 (2.5)	0.024		
Azithromycin	9 (1.1)	130 (0.4)	0.603		
Clarithromycin	0 (0.0)	15 (0.1)	0.351		
Ceftriaxone	14 (1.7)	126 (0.9)	0.018		
Levofloxacin	12 (1.5)	204 (1.4)	0.959		
Ciprofloxacin	54 (6.5)	1273 (8.9)	0.018		
Fluoroquinolones	66 (8.0)	1477 (10.4)	0.029		
Metronidazole	12 (1.5)	257 (1.8)	0.460		
Vancomycin	1 (0.1)	11 (0.1)	0.664		
Doxycycline	3 (0.4)	27 (0.2)	0.276		

EMS: emergency medical service, Hb: hemoglobin, WBC: white blood cell, CRP: C-reactive protein, GERD: gastroesophageal reflux disease, IBD: inflammatory bowel disease, COPD: chronic obstructive pulmonary disease.

**Table 4:** Determinants of ED readmission within seven days in patients aged over 65 years old.

Variable	Readmission within 7 Days		Univariate *p* Value	Odds Ratio [95% Confidence Interval]	Multivariate *p* Value
	YES (N 391)	NO (N 5127)			
Sex (male)	171 (43.7)	1901 (37.1)	0.009	1.291 [1.047–1.592]	0.017
Age (years)	75 (70–81)	76 (70–82)	0.55		
Triage code					
Emergency	2 (0.5)	56 (1.1)			0.337
Urgent	121 (30.9)	1884 (26.7)	0.032	1.702 [0.409–7.076]	0.465
Non-urgent	268 (68.5)	3187 (62.2)		2.029 [0.490–8.404]	0.329
Access to EMS	83 (21.2)	1364 (26.6)	0.020	0.861 [0.663–1.119]	0.264
Symptoms > 24 h	225 (57.5)	2480 (48.4)	0.000	1.299 [1.041–1.622]	0.021
Length of stay in the ED (hours)	8.6 [5.1–21.1]	9.0 [5.5–24.0]	0.102		
Refusal of hospitalization	62 (15.9)	599 (11.7)	0.014	1.438 [1.077–1.918]	0.014
Associated complaints					
Fever	143 (36.6)	1514 (29.5)	0.003	1.336 [1.072–1.665]	0.010
Vomiting	237 (60.6)	3109 (60.6)	0.992		
Nausea	124 (31.7)	1723 (33.6)	0.445		
Abdominal pain	219 (56)	2646 (51.6)	0.093		
Chest pain	18 (4.6)	299 (5.8)	0.314		
Syncope	33 (8.4)	659 (12.9)	0.011	0.682 [0.467–0.996]	0.047
Melena	5 (1.3)	77 (1.5)	0.725		
Rectorrhagia	10 (2.6)	236 (4.6)	0.059		
Belching/hiccups	3 (0.8)	26 (0.5)	0.493		
Malaise	68 (17.4)	952 (18.6)	0.563		
Laboratory parameters					
Hb (g/dL)	13.2 [11.7–14.4]	13.1 [11.9–14.2]	0.716		
WBC (cell/mm^3^)	8.08 [5.81–10.89]	8.50 [6.70–10.90]	0.177		
Creatinine (mg/dL)	1.04 [0.80–1.37]	0.90 [0.71–1.17]	0.003		
Blood glucose (mg/dL)	116 (96–131)	115 (100–138)	0.537		
Procalcitonin (ng/dL)	0.06 [0.05–0.15]	0.05 [0.05–0.11]	0.693		
CRP (mg/L)	11.9 [2.3–43.0]	9.5 [1.9–34.5]	0.596		
Clinical history					
Diverticulosis	27 (6.9)	536 (10.5)	0.025	0.601 [0.401–0.901]	0.014
GERD	10 (2.6)	145 (2.8)	0.755		
IBD	5 (1.3)	71 (1.4)	0.862		
Cardiac disease	63 (16.1)	876 (17.1)	0.621		
COPD	27 (6.9)	337 (6.6)	0.799		
Diabetes	65 (16.6)	875 (17.1)	0.822		
Chronic renal failure	15 (3.8)	156 (3.0)	0.383		
Liver disease	7 (1.8)	88 (1.7)	0.914		
Dementia	12 (3.1)	223 (4.3)	0.227		
Prescription at discharge					
No antibiotics/probiotics	294 (75.2)	3885 (75.8)	0.931		
Only antibiotics	81 (20.7)	1031 (20.1)	0.773	0.974 [0.7491.266]	0.843
Antibiotics+probiotics	5 (1.3)	82 (1.6)	0.624	0.781 [0.312–1.955]	0.598
Only probiotics	11 (2.8)	129 (2.5)	0.719	1.105 [0.587–2.081]	0.757
Amoxicillin	0 (0.0)	12 (0.2)	0.338		
Amoxicillin/clavulanic acid	8 (2.0)	101 (2)	0.917		
Azithromycin	2 (0.5)	58 (1.1)	0.255		
Clarithromycin	0 (0.0)	6 (0.1)	0.499		
Ceftriaxone	7 (1.8)	7.9 (1.5)	0.701		
Levofloxacin	6 (1.5)	93 (1.8)	0.688		
Ciprofloxacin	25 (6.4)	377 (7.4)	0.482		
Fluoroquinolones	31 (7.9)	470 (9.2)	0.411		
Metronidazole	9 (2.3)	126 (2.5)	0.848		
Vancomycin	1 (0.3)	8 (0.2)	0.638		
Doxycycline	0 (0.0)	1 (0)	0.782		

EMS: emergency medical service, Hb: hemoglobin, WBC: white blood cell, CRP: C-reactive protein, GERD: gastroesophageal reflux disease, IBD: inflammatory bowel disease, COPD: chronic obstructive pulmonary disease.

## Data Availability

All data are included within the main text.
